# Initiation of a *Helicobacter pylori* Screening Program: Enhancing Healthcare at Juntendo University

**DOI:** 10.14789/jmj.JMJ23-0043-OA

**Published:** 2024-05-24

**Authors:** KUMIKO UEDA, MARIKO HOJO, KANAMI ITO, SHOTARO OKI, TSUTOMU TAKEDA, YOICHI AKAZAWA, HIROYA UEYAMA, HIROSHI FUKUDA, TOSHIO NAITO, AKIHITO NAGAHARA

**Affiliations:** 1 Department of Gastroenterology, Juntendo University Faculty of Medicine, Tokyo, Japan; 1 Department of Gastroenterology, Juntendo University Faculty of Medicine, Tokyo, Japan; 2 Health & Safety Promotion Center, Juntendo University Faculty of Medicine, Tokyo, Japan; 2 Health & Safety Promotion Center, Juntendo University Faculty of Medicine, Tokyo, Japan; 3 Department of General Medicine, Juntendo University Faculty of Medicine, Tokyo, Japan; 3 Department of General Medicine, Juntendo University Faculty of Medicine, Tokyo, Japan

**Keywords:** *Helicobacter pylori*, screening, eradication, university students

## Abstract

**Objectives:**

We started *Helicobacter pylori* (*H. pylori*) screening program of students at Juntendo university in 2020. We report the current status of *H. pylori* screening program and the outcomes of *H. pylori* screening program.

**Methods:**

The students of the School of the Faculty of Health Sciences of Juntendo University enrolling in the spring of 2020-2022 were recruited for this study. The anti-*H. pylori* antibody test was used for detecting *H. pylori* infection. An individual with a serum anti-*H. pylori* antibody titer of less than 3 U/ml was considered to be negative for *H. pylori* infection. If the antibody titer was 3 U/ml or higher, the subject was considered to be possibly infected and recommended to visit a hospital for further testing. Esophagogastroduodenoscopy and 13C urea breath test were performed for diagnosing *H. pylori* infection at the hospital. Eradication therapy was performed, and the eradication assessment were performed at least 8 weeks after the end of eradication therapy.

**Results:**

Seven hundred twenty-eight students were screened for *H. pylori* from 2020 to 2022. Fifty-seven students were recommended to visit a hospital based on the anti-*H. pylori* antibody serum test. Forty-seven students visited Juntendo university hospital. Eleven of the 47 students were positive for *H. pylori* and all of them students received eradication therapy. *H. pylori* eradication was successful in nine of the 11 students.

**Conclusions:**

The *H. pylori* screening program for university students at Juntendo university has been successfully initiated with nine successful eradications since its inception in 2020.

## Introduction

*Helicobacter pylori* (*H. pylori*) infection is primarily acquired during childhood and can persist for many years if left untreated^1, 2)^. Although many people infected with *H. pylori* do not experience any symptoms, it can cause a variety of gastrointestinal problems including gastritis, peptic ulcers, and increased risk of gastric cancer^1, 3)^.

*H. pylori* eradication therapy for young people has been shown to be effective as a measure to prevent gastric cancer^4, 5)^. It is important to diagnose whether individuals are infected with *H. pylori* and to provide *H. pylori* eradication therapy to infected individuals.

We have initiated a *H. pylori* screening program to detect infection and facilitate its treatment in 2020. Now that several years have passed, we report the current status of *H. pylori* screening program at Juntendo University and the outcomes of *H. pylori* screening program.

## Methods

### Study design

This study was a retrospective cohort study. The protocol used for this study was reviewed and approved by the Institutional Ethics Committee of the Juntendo University (Approval number: E22- 0063).

### Subjects

The students of the School of the Faculty of Health Sciences of Juntendo University enrolling in the spring of 2020-2022 were recruited for this study.

### Methods

#### The method for *Helicobacter pylori* screening

##### 1. Explanation of *H. pylori* screening to the subjects

*H. pylori* screening instruction manual before *H. pylori* screening were distributed to the students. The instruction manual included information about *H. pylori* infection, the significance of screening, symptoms of infection, screening methods, how to read the screening results, treatment methods, and the benefits of performing *H. pylori* eradication therapy (Supplementary Figure 1).

It was explained that students who do not wish to undergo *H. pylori* screening can refuse to undergo the *H. pylori* screening.

The handout was given to the students with the result of *H. pylori* screening which explains the relationship between *H. pylori* infection and gastric cancer, and the effect of *H. pylori* eradication therapy on the prevention of gastric cancer in different age groups. *H. pylori* eradication therapy has been shown to be highly effective in preventing *H. pylori* infection in the next generation^6)^ (Supplementary Figure 2).

##### 2. Methods of detecting *H. pylori* infection and criteria for hospital referral

**Figure 1:**
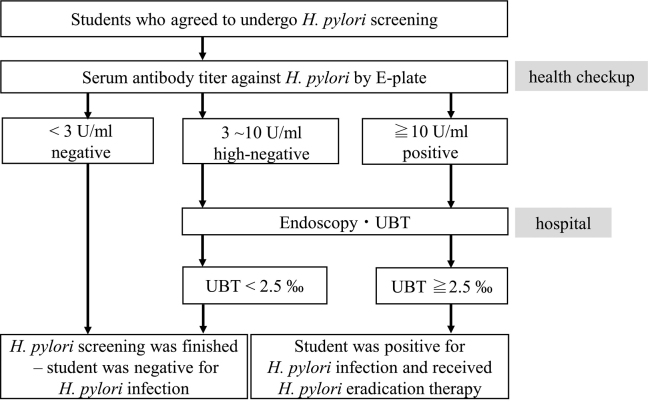
Flowchart of *H. pylori* screening program at Juntendo university We recommended that students with a serum antibody titer of 3 U/ml or higher be examined at a hospital. They underwent EGD and additional examinations such as the UBT for diagnosing *H. pylori* infection at the hospital. A value of 2.5‰ or above on the UBT indicates that the individual is positive for *H. pylori* infection and should be treated with *H. pylori* eradication therapy. *H. pylori*, *Helicobacter pylori*; E-plate, method of detecting serum *H. pylori* antibody; UBT, 13C-urea breath test; EGD, esophagogastroduodenoscopy

The anti-*H. pylori* antibody test (E-plate Ⅱ ‘Eiken’ *H. pylori* antibody; Eiken Chemical Co., Ltd., Tochigi, Japan)^7)^was used for detecting *H. pylori* infection. Blood samples were obtained during the health check upon admission of students to the school. An individual with a serum anti-*H. pylori* antibody titer of less than 3 U/ml was considered to be negative for *H. pylori* infection. An anti-*H. pylori* antibody titer of 3-10 U/ml was defined as high-negative titer, and that of more than 10 U/ml was defined as positive titer (Figure 1). If the antibody titer was 3 U/ml or higher, the subject was considered to be possibly infected and recommended to visit a hospital for further testing.

#### Hospital Evaluation and Follow-up Protocols

Esophagogastroduodenoscopy (EGD) and 13C urea breath test (UBT, Otsuka Pharmaceutical Co., Ltd., Tokyo, Japan) were performed for diagnosing *H. pylori* infection at the hospital. A result of 2.5‰ or above on the UBT was defined as UBT-positive for *H. pylori* infection. *H. pylori* eradication therapy was recommended for individuals who are positive for *H. pylori* infection by the UBT. Students with a result of less than 2.5‰ on the UBT were defined free of *H. pylori* infection, and they were deemed unnecessary for further medical attention (Figure 1).

#### Eradication therapy and assessment of *H. pylori* eradication

Eradication therapy consisted of 20 mg vonoprazan, 750 mg amoxicillin, and 200 mg clarithromycin twice a day for 7 days. UBT were performed for the eradication assessment at least 8 weeks after end of therapy.

## Results

**Figure 2:**
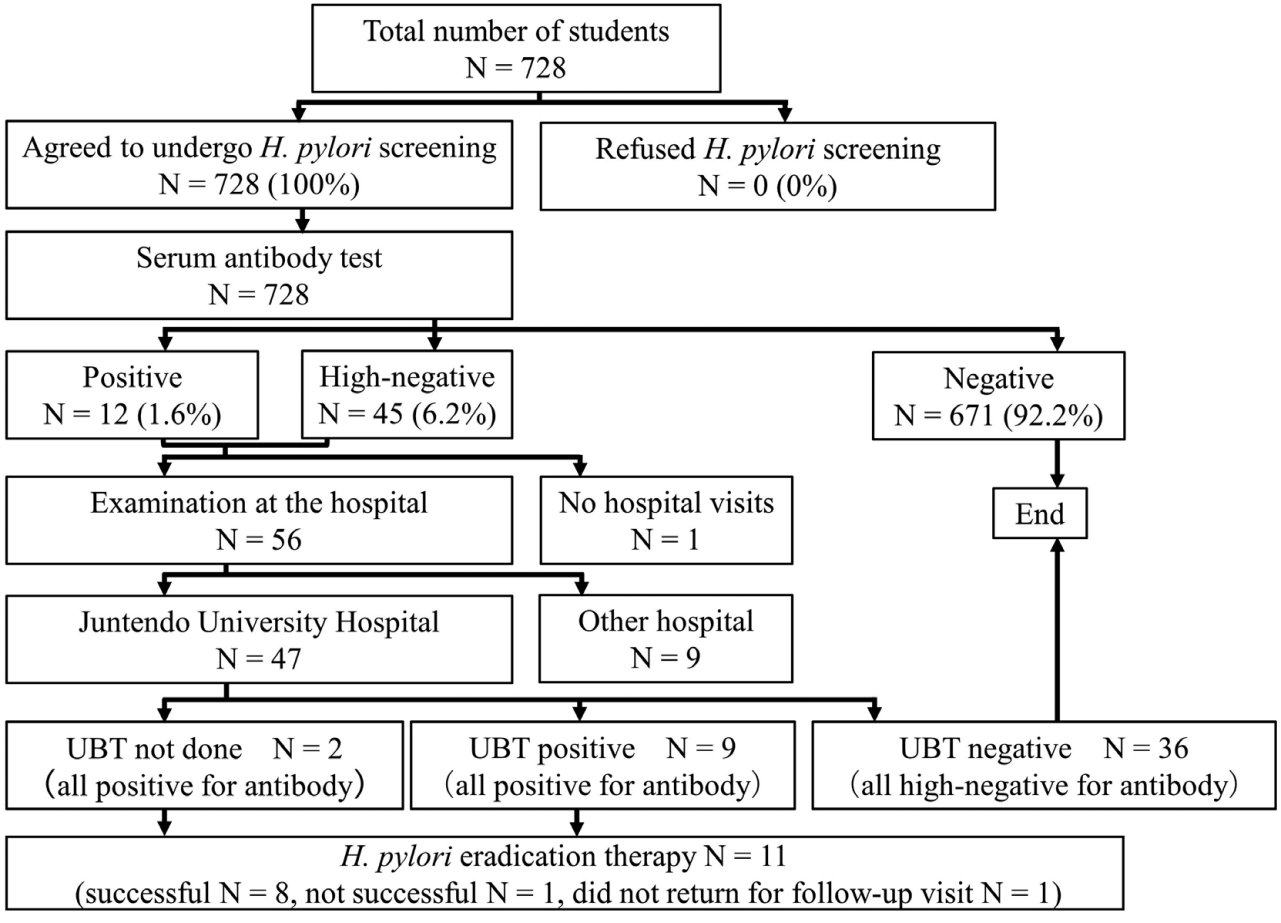
Summary of the results of our *H. pylori* screening program in three incoming classes of students A total of 728 people in the three incoming classes of students were eligible for screening for *H. pylori*, and all 728 individuals (100%) agreed to undergo screening for *H. pylori* infection. Twelve individuals (1.6%) had a positive titer, while 45 individuals (6.2%) had a high-negative titer. Fifty-six of the 57 students with a positive or high-negative titer visited a hospital for further testing. Forty-seven students visited Juntendo University Hospital, and eleven of those who visited Juntendo University Hospital were positive for *H. pylori* infection and received *H. pylori* eradication therapy. Among the 11 students who underwent *H. pylori* eradication therapy, *H. pylori* was successfully eradicated in 9 students.

The number of first-year students of the School of Faculty of Health Sciences who were screened for *H. pylori* from 2020 to 2022 was 728. No student refused to participate in the screening, and the participation rate in *H. pylori* screening among the three incoming classes of students was 100%. Twelve students (1.6%) had a positive titer and 45 students (6.2%) had a high-negative titer. Therefore, a total of 57 students (7.8％ of total) were recommended to visit a hospital for further testing. Of these, fifty-six students visited a hospital. Only one student who had a high-negative titer did not visit a hospital. Forty-seven out of the 56 students visited Juntendo University Hospital. Nine of the 47 students were positive for *H. pylori* on the UBT. These nine students also had positive titers, and all of them underwent *H. pylori* eradication therapy. Two of the 47 students did not undergo UBT based on each doctor’s decision. The two students had also positive titer and all of them students underwent *H. pylori* eradication therapy. All students with high-negative titers for serum *H. pylori* antibody were negative on the UBT. *H. pylori* eradication therapy was successful in nine of the 11 students, and it was unsuccessful in one student. The remaining one *H. pylori*-positive student did not visit the hospital after receiving prescriptions for the *H. pylori* eradication therapy (Figure 2). Of the nine students who visited other hospitals, one had a positive titer and underwent eradication therapy. The results of eradication assessment of were not interviewed. Eight of the subjects had a high-negative titer, and all of them are under observation.

## Discussion

This report summarized the current status of *H. pylori* screening program held at Juntendo University and the outcomes of the first three years of the screening. *H. pylori* eradication therapy is known to be effective in reducing the risk for gastric cancer^4, 5, 8)^. Therefore, we started *H. pylori* screening program of students to reduce the risk of gastric cancer among Juntendo University students.

No student refused to participate in the screening, and participation rates in the *H. pylori* screening were high from the first year and continued to be high in following years. All but one of the students who were recommended to visit a hospital during the three-year period, even though they had no symptoms, visited a hospital for follow-up testing and treatment, and the initiative completion rate was 98% (56/57). First, we distributed an instruction manual for *H. pylori* screening before *H. pylori* screening (Supplementary Figure 1). The handout explaining the interpretation of the results was distributed to the students with the result of *H. pylori* screening (Supplementary Figure 2).

We believe that our educational activities to inform students of the significance of *H. pylori* screening have been successful. Also, the fact that the target groups of our *H. pylori* screening program were students, who were easy to follow, was one factor in the extreme high initiative completion rate.

Our *H. pylori* screening program is intended for university students, who range in age from 18 to 30 years old. Most of the target population are young. Some reports suggest that eradication therapy is effective at all ages^4, 9)^. Whether or not *H. pylori* screening will reduce the number of gastric cancer cases, reduce the number of *H. pylori*-infected individuals in the next generation, and reduce the incidence of *H. pylori*-related diseases such as gastroduodenal ulcers, will become evident through long- term observation of the outcomes following the initiation of this program. Furthermore, we believe that continuing *H. pylori* screening program not only will lead to a lower risk of gastric cancer and a reduction in the number of people infected with *H. pylori* in the next generation, but also will provide information on the incidence of *H. pylori* infection, especially among young people.

The positive predictive value of the urinary anti- *H. pylori* antibody tests was 61.2%, suggesting the presence of false positives^10)^; therefore, we measured the serum antibody titer against *H. pylori*. With the E-plate Ⅱ ‘Eiken’ *H. pylori* antibody test, uninfected subjects have a test result of less than 3 U/ml, but a small number of individuals with present infection have a high-negative titer^11, 12)^. Therefore, we recommended that individuals with serum antibodies of 3 U/ml or higher visit a hospital for further testing and treatment for positive cases. However, in this study, 36 high-negative students were UBT negative. Toyoshima et al. reported that 17% of cases with high-negative titers between 3 U/ml and 10 U/ml were positive on the UBT^11)^. The positive rate on the UBT among individuals with an antibody titer of less than 3 U/ml is reported to be 0.3%^12)^, and we decided to continue to recommend that individuals with an antibody titer of 3 U/ml or higher visit a hospital for further testing using a test method with high sensitivity for a few more years. According to future results, we might raise the antibody titer threshold at which we recommend that individuals visit a hospital for further testing. We began targeting first-year university students, and we have not been asking individuals whether they had a history of receiving *H. pylori* eradication therapy in the past. However, there are already reports of young patients receiving *H. pylori* eradication therapy^13-16)^, and some university students may have already received *H. pylori* eradication therapy. In order to avoid unnecessary tests in the future, it is necessary to interview individuals to determine whether they had received *H. pylori* eradication therapy in the past. Follow-up strategies for those who were detected *H. pylori* infection and have been treated with eradication have not yet been determined. This matter represents a crucial area for future research.

In conclusion, the *H. pylori* screening program for university students at Juntendo University has been successfully initiated, with high participation rates since its inception in 2020. A longer observation period is needed to assess whether this screening program can reduce the gastric cancer rate and whether it can reduce the number of *H. pylori*- infected individuals in the next generation. We should further expand the target population and examine the results in the future.

## Funding

The authors received no financial support for the research.

## Author contributions

KU and AN designed the study. KU, KI, HF, and TN considered of operational methods. KI prepared for performing the study. KU analyzed and interpreted the data. KU and MH drafted and revised the manuscript. KU, SO, TT, YA and HU conducted a medical examination on the target patient. All authors reviewed the manuscript and approved the final version of the manuscript.

## Conflicts of interest statement

The authors declare that there are no conflicts of interest.

## Supplementary Material

Supplementary Figure 1.

Supplementary Figure 2.
