# Supplementary figures and images for: Initiation of a Helicobacter pylori Screening Program: Enhancing Healthcare at Juntendo University
Source: Juntendo Iji Zasshi. 2024 May 24;70(3):214–20. doi: 10.14789/jmj.JMJ23-0043-OA (PMC11487367; doi:10.14789/jmj.JMJ23-0043-OA)

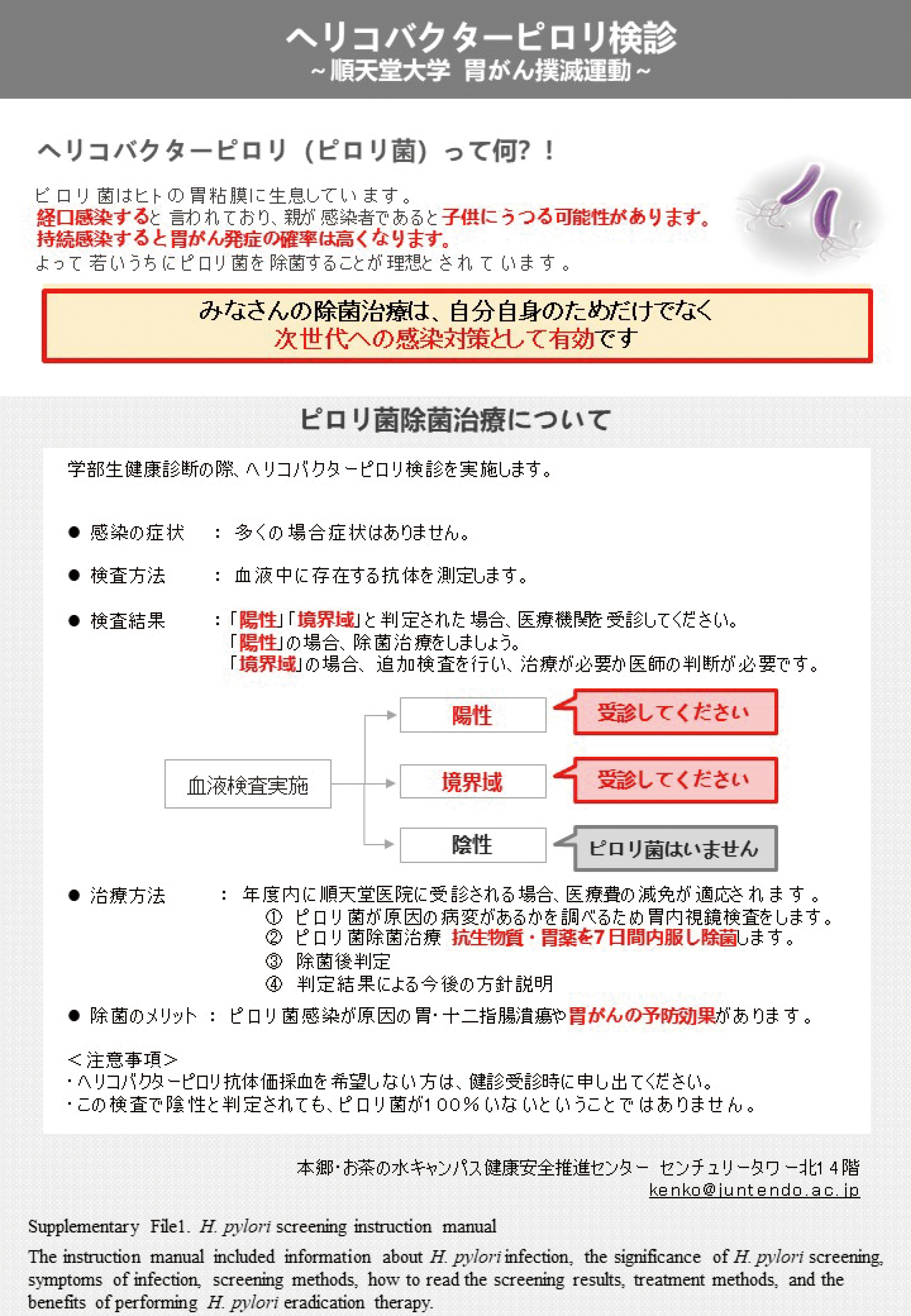

Supplement: Supplementary Figure 1. [file 2188-2126-70-3-0214-s001.jpg]

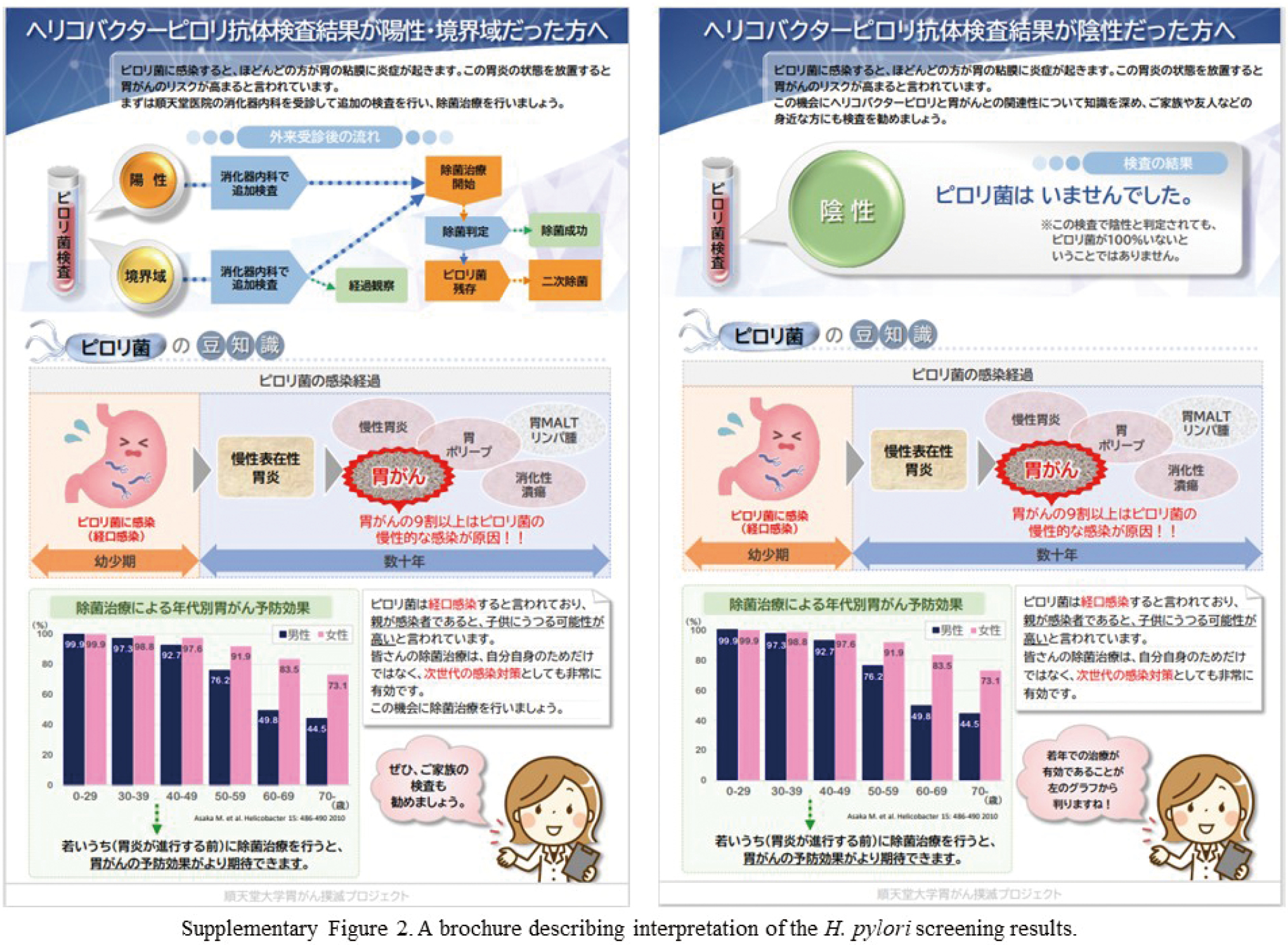

Supplement: Supplementary Figure 2. [file 2188-2126-70-3-0214-s002.jpg]
